# Oral cleaning habits and the copy number of periodontal bacteria in pregnant women and its correlation with birth outcomes: an epidemiological study in Mibilizi, Rwanda

**DOI:** 10.1186/s12903-022-02443-4

**Published:** 2022-09-26

**Authors:** Hiroaki Arima, Akintije Simba Calliope, Hideki Fukuda, Theoneste Nzaramba, Marie Goretti Mukakarake, Takayuki Wada, Takashi Yorifuji, Leon Mutesa, Taro Yamamoto

**Affiliations:** 1 Department of International Health and Medical Anthropology, Institute of Tropical Medicine, Nagasaki University, 1-12-4 Sakamoto, Nagasaki, 852-8523 Japan; 2 Kishokai Medical Corporation, Aichi, Japan; 3 College of Medicine and Health Sciences, University of Rwanda, Kigali, Rwanda; 4 National Institute of Public Health, Saitama, Japan; 5 Mibilizi Hospital, Rusizi, Rwanda; 6 Graduate School of Human Life and Ecology, Osaka Metropolitan University, Osaka, Japan; 7 Department of Epidemiology, Graduate School of Medicine, Dentistry and Pharmaceutical Sciences, Okayama University, Okayama, Japan; 8 Department of Human Genetics, College of Medicine and Health Sciences, University of Rwanda, Kigali, Rwanda

**Keywords:** Periodontal bacteria, Rwanda, Pregnant women, Low birth weight, Preterm birth

## Abstract

**Background:**

Since 1996, many studies have reported that periodontal disease during pregnancy may be a risk factor for preterm birth and low birth weight; however, in Africa, periodontal disease is considered a non-high-priority disease. In addition, there are few dental facilities in rural Rwanda; thus, the oral condition of pregnant women has not been investigated. The objective of this study was to assess the tooth brushing habits of pregnant women in rural Rwanda and evaluate whether periodontal bacteria in the oral cavity of pregnant women are related to birth outcomes or oral cleaning habits.

**Methods:**

A questionnaire survey and saliva collection were conducted for pregnant women in the catchment area population of Mibilizi Hospital located in the western part of Rwanda. Real-time PCR was performed to quantitatively detect total bacteria and 4 species of periodontal bacteria. The relationship of the copy number of each bacterium and birth outcomes or oral cleaning habits was statistically analyzed.

**Results:**

Among the participants, high copy numbers of total bacteria, *Tannerella forsythia*, and *Treponema denticola* were correlated with lower birth weight (*p* = 0.0032, 0.0212, 0.0288, respectively). The sex ratio at birth was higher in women who had high copy numbers of *Porphyromonas gingivalis* and *T. denticola* during pregnancy (*p* = 0.0268, 0.0043). Furthermore, regarding the correlation between oral cleaning habits and the amount of bacteria, the more frequently teeth were brushed, the lower the level of *P. gingivalis* (*p* = 0.0061); the more frequently the brush was replaced, the lower the levels of *P. gingivalis* and *T. forsythia* (*p* = 0.0153, 0.0029).

**Conclusions:**

This study suggested that improving tooth brushing habits may reduce the risk of periodontal disease among pregnant women in rural Rwanda. It also indicated that the amount of bacteria is associated with various birth outcomes according to the bacterial species. Both access to dental clinics and the oral cleaning habits of pregnant women should be important considerations in efforts to alleviate reproductive-related outcomes in rural Africa.

**Supplementary Information:**

The online version contains supplementary material available at 10.1186/s12903-022-02443-4.

## Plain English summary

Periodontal disease is known to cause many complications. For instance, pregnant women with periodontal disease are at increased risk of preterm birth and delivering low-birth-weight infants. However, the importance of oral care during pregnancy is not an important consideration in rural Africa, where preterm birth rates and low-birth-weight rates are particularly high. Moreover, even the oral hygiene status of pregnant women has not been assessed in such areas. In this study, we focused on the amount of periodontal bacteria that cause periodontal disease and investigated the relationship between the amount of bacteria and birth outcomes. Our findings indicate that tooth brushing guidance for pregnant women and improved access to dental clinics in rural Africa may contribute to reduced rates of preterm birth and low birth weight.

**Supplementary Information:**

The online version contains supplementary material available at 10.1186/s12903-022-02443-4.

## Background

For many years, periodontal disease was treated only in the dental field; however, in 1996, Offenbacher et al. reported for the first time that periodontal disease during pregnancy may induce preterm birth and low birth weight [1]. Since then, there have been many investigations regarding the impact of periodontal disease on pregnancy. For instance, in pregnant women with periodontal disease, inflammatory cytokines, such as interleukin-1β (IL-1β), interleukin-6 (IL-6), and prostaglandin E2 (PGE2), which are produced by stimulated periodontal tissue, reach the placenta and fetus through blood flow. Such cytokines cause placental inflammation and fetal stunting, resulting in premature birth and low birth weight [2]. In fact, the levels of IL-6 and PGE2 in amniotic fluid have been reported to be higher in pregnant women with periodontal disease during pregnancy than in those without periodontal disease during pregnancy [3]. In addition, studies targeting bacterial mass indicate that the severity of periodontal disease is correlated with the amount of periodontal bacteria in the oral cavity [4] and that the amount of periodontal bacteria is associated with the birth outcome according to the bacterial species [5]. Thus, many organizations, including the US Centers for Disease Control and Prevention and the European Periodontal Disease Federation, have declared that oral care during pregnancy is more important for preventing preterm birth and low birth weight than initially thought [6, 7]. However, the condition of the oral cavity of pregnant women as well as the relationship between the copy number of periodontal bacteria and birth outcomes have only been assessed in developed countries, not in developing countries, including rural Rwanda, because there are very few resources or interest in this type of research. Furthermore, it has been suggested that periodontal bacteria may be detected around teeth and implants even before signs of inflammation appear in the periodontal tissue. Thus, it is possible to widely evaluate the oral conditions of pregnant women up to the predisease stage. In addition, metagenomic analysis and 16S rRNA gene analysis have contributed greatly to clarifying the characteristics of the oral flora [8]. It has also been reported that the diversity of the oral flora differs depending on ethnicity [9]. Therefore, to evaluate the risk of preterm birth and low birth weight in Rwanda, we quantitatively detected the amount of periodontal bacteria in the oral cavities of pregnant women in Rwanda. Thus, the objective of this study was to investigate the tooth brushing habits of pregnant women and determine whether the copy number of periodontal bacteria in the oral cavity is related to birth outcomes in rural Rwanda. We also aimed to identify whether the copy number of bacteria is actually affected by oral cleaning habits. Based on these analyses, we broadly examined whether inadequate tooth brushing habits of pregnant women in rural Rwanda increased the risk of periodontal disease and whether it had a negative impact on pregnancy outcomes.

## Methods

### Field survey

**Fig. 1:**
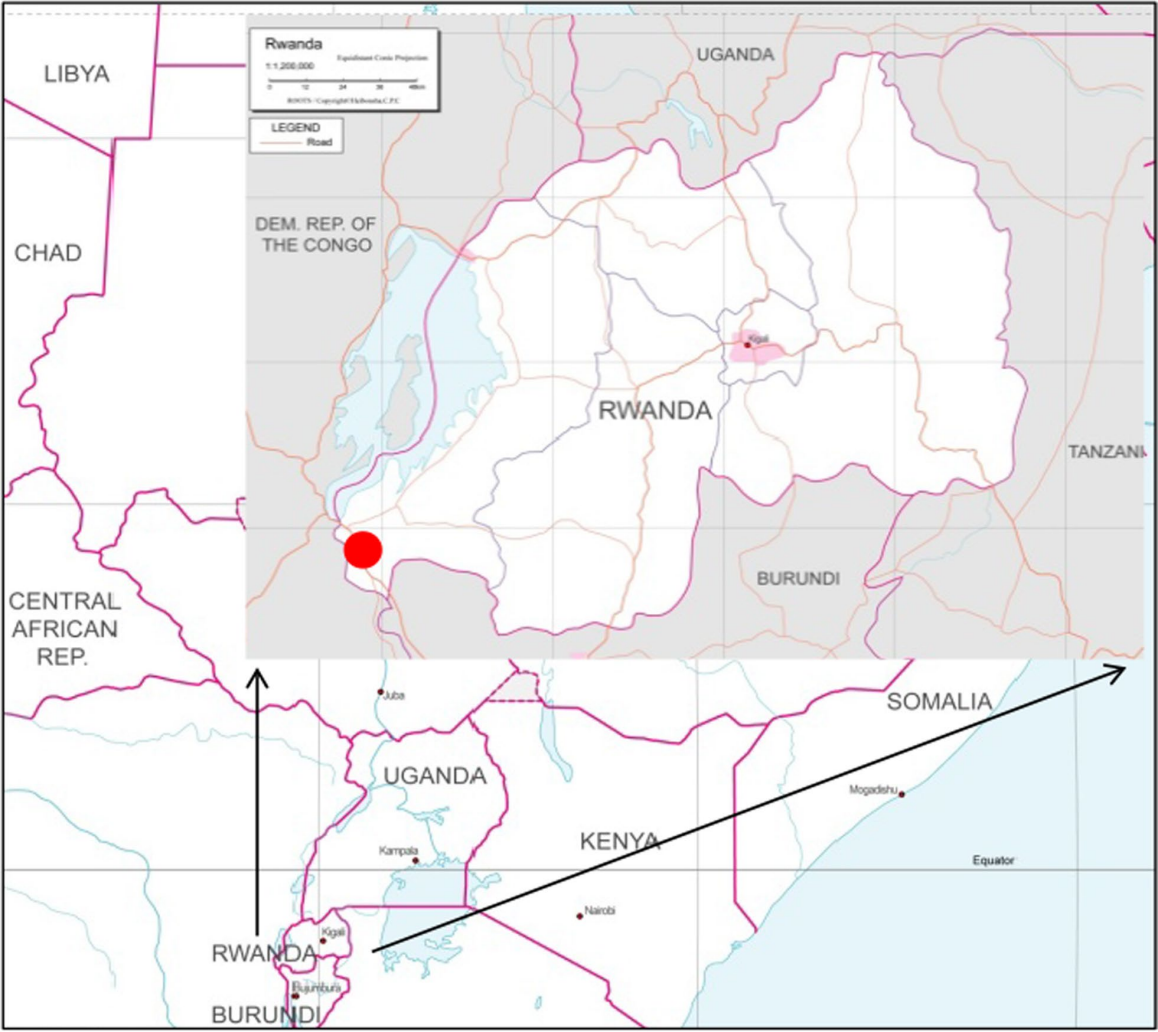
Geographical location of the survey site. The survey site, Mibilizi (Red dot), is located near the border with the Democratic Republic of the Congo, and approximately 240 km from the capital Kigali

A survey was conducted with pregnant women who were outpatients or hospitalized at Mibilizi Hospital in the Rusizi district, western Rwanda (Fig. 1). Pregnant women aged 16 years or older who signed a consent form were included, regardless of their gestational age at the participation. Pregnant women who were hospitalized due to factors other than perinatal matters, those who were hospitalized for threatened premature labor, and those who were ready to deliver were excluded from this study. Pregnant women who gave birth to twins were also excluded from the analysis to appropriately evaluate the effects of periodontal bacteria on birth weight. Nurses interviewed the pregnant women using questionnaires to collect data on basic attributes, pregnancy status, and oral cleaning habits [10]. In addition, to detect periodontal bacterial DNA in the oral cavity of the pregnant women, a saliva sample was collected with DNA/RNA Shield (Zymo Research, CA, USA) and correctly stored until further analysis. After the participants gave birth, a follow-up survey was conducted to obtain data on the gestational age, birth weight, birth length, and sex of the newborn.

### Detecting periodontal bacterial DNA

Genomic DNA was extracted from 200 μl of saliva using the QIAamp DNA Mini Kit (Qiagen, Hilden, Germany), and the DNA of periodontal bacteria was quantitatively detected by real-time PCR using a LightCycler 480 (Roche, Basel, Switzerland). We targeted 4 species, including 3 “red complex” species (*Porphyromonas gingivalis*, *Tannerella forsythia* and *Treponema denticola*) and *Prevotella intermedia*. Specific primers (final concentration of 500 nM) against each 16S rRNA region and probes labeled with FAM at the 5' end and TAMURA at the 3' end (final concentration of 200 nM) were used. We also performed real-time PCR using universal primers targeting the common bacterial 16S rRNA sequence to evaluate the copy number of total bacteria in the saliva (Additional file 1: Table S1). FastStart TaqMan Probe Master Mix (Roche, Basel, Switzerland) for specific periodontal bacteria and LightCycler 480 SYBR Green I Master Mix (Roche, Basel, Switzerland) for total bacteria were used as premix reagents. The amount of DNA of each periodontal bacterial species in the sample was calculated from the calibration curve prepared using control DNA and the Ct value obtained by real-time PCR. In addition, the copy number of each bacterial species was calculated by dividing the weight of detected DNA by the weight of the DNA corresponding to the genome size per copy [11]. In this study, DNA extracted from *P. gingivalis* ATCC33277, *T. forsythia* ATCC43037, *T. denticola* ATCC35405 and *P. intermedia* ATCC25611 was used as a positive control for detection. The DNA of *P. gingivalis* was also used as a positive control for detecting total bacteria [12]. The PCR reaction conditions for targeting *P. gingivalis*, *T. forsythia* and *T. denticola* were 95 °C for 1 min, 50 cycles of 95 °C for 5 s, 57 °C for 15 s, and 72 °C for 5 s, and a final cooling at 40 °C for 8 min*.* The conditions for detecting *P. intermedia* DNA were 95 °C for 1 min, 50 cycles of 95 °C for 5 s, 56 °C for 15 s, and 72 °C for 8 s, and a final cooling at 40 °C for 8 min. PCRs using universal primers to detect total bacteria were performed at 95 °C for 1 min, 50 cycles of 95 °C for 5 s, 58 °C for 15 s, and 72 °C for 20 s, and a final cooling at 40 °C for 8 min [12].

### Statistical analysis

The data from the questionnaire survey were summarized descriptively, and Spearman's rank correlation coefficient, Fisher's exact test, Welch’s t test, Wilcoxon's rank sum test, the Cochrane Armitage test, linear regression analysis, and a generalized linear model were used for statistical analysis. A *p* value of < 0.05 was considered significant, and R (version 3.5.3) and R studio were used for the statistical analyses.

## Results

### Characteristics of the target pregnant women

In all, 153 women who had a single fetus were included in this study. The average age of the pregnant women was 31.2 ± 6.5 years, and the average gestational age at participation was 27.7 ± 3.8 weeks (Table 1). A total of 26.1% (n = 40) of the participants were pregnant for the first time, and 17.0% (n = 26) were pregnant for at least the sixth time.

**Table 1:** Characteristics of the participants

Average age	31.2 ± 6.5
Age group	
< 18	1 (0.7)
18–35	104 (68.0)
> 35	48 (31.4)
Average gestational age (weeks)^a^	27.7 ± 3.8
Gravidity	
First	40 (26.1)
2–3 times	51 (33.3)
4–5 times	36 (23.5)
6–7 times	19 (12.4)
8 times or more	7 (4.6)

Data from 153 participants were compiled. Values indicate the average ± SD or n (%)

^a^Data on gestational age are missing for 3 women

### Carrier status regarding periodontal bacteria

**Fig. 2:**
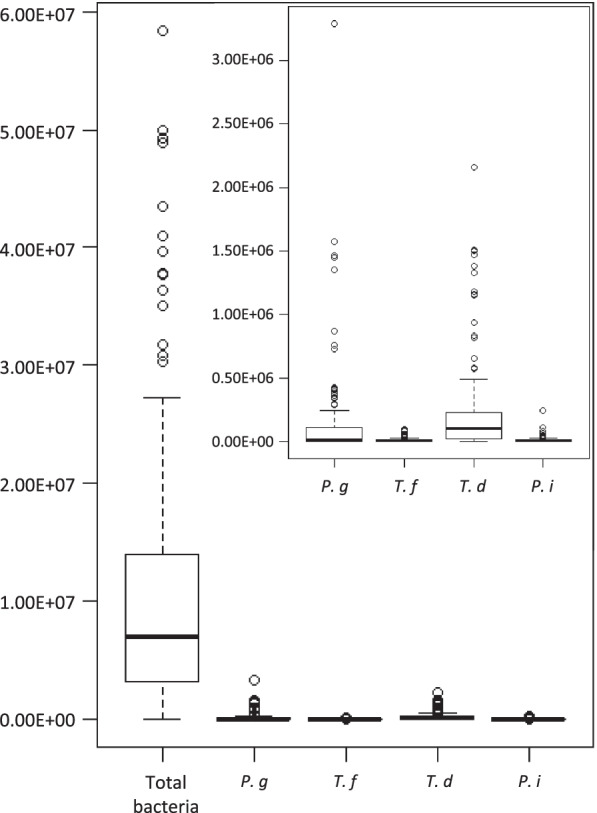
Copy number of each bacteria detected from the participant's saliva. This figure shows the distribution of each copy number of bacteria. The figure on the upper right is a comparison of periodontal bacteria only

The positive rate of periodontal bacteria among the pregnant women was 97.4% (n = 149) for *T. forsythia*, 96.1% (n = 147) for *T. denticola*, and 93.5% (n = 143) for *P. intermedia* and was the lowest at 58.8% (n = 90) for *P. gingivalis*. Only 1.3% (n = 2) of the pregnant women did not have any of the 4 species of periodontal bacteria (Additional file 2). The median copy number of each bacterium (1st quartile–3rd quartile) was 7.01E7 (3.19E6 –1.40E7) for total bacteria, 1.56E4 (0.00 –1.12E5) for *P. gingivalis*, 5.92E3 (1.41E3–1.22E4) for *T. forsythia*, 1.00E5 (1.88E4 –2.32E5) for *T. denticola* and 3.13E3 (6.47E2 –1.05E4) for *P. intermedia* in positive subjects (Fig. 2). Wilcoxon's rank sum test showed that the copy number of *T. denticola* was significantly higher than that of the other three species (vs. *P. gingivalis*: *p* < 0.0001; vs. *T. forsythia*: *p* < 0.0001; vs. *P. intermedia*: *p* < 0.0001).

### Birth outcomes for the participants

Since twin fetuses generally tend to be born smaller than single fetuses, the data from 153 fetuses, excluding the twin fetuses from 3 pregnant women, were used for statistical analysis. Table 2 summarizes the birth outcomes for the participants. Of the newborns, 67 (43.8%) were male and 86 (56.2%) were female, and the sex ratio at birth (male births per female births) tended toward being female. Overall, the preterm birth rate was 2.6% (n = 4), and the rate of low birth weight and very low birth weight was 6.0% (n = 9). Regarding birth weight, 2000 g or more and less than 2500 g was defined as low birth weight, and 1500 g or more and less than 2000 g was defined as very low birth weight. There were no significant differences in the gestational age, birth weight, or birth length by sex among the newborns.

**Table 2:** Summary of the participants’ birth outcomes

	Total (n = 153)	Male (n = 67)	Female (n = 86)	*p* value
Delivery period^a^				
Preterm birth (< 37 weeks)	4 (2.6)	2 (3.0)	2 (2.3)	0.6146
Full-term birth (37 ≤ GA < 41 weeks)	147 (96.7)	63 (95.5)	84 (97.7)	
Post-term birth (≥ 42 weeks)	1 (0.7)	1 (1.5)	0 (0.0)	
Average birth weight(g)^b^	3210.5 ± 529.6	3303.0 ± 511.7	3138.7 ± 535.1	0.0570
Birth weight^b^				
Normal (≤ 2500 g)	142 (94.0)	63 (95.5)	79 (92.9)	0.3696
Low birth weight (2000 g ≤ BW < 2500 g)	6 (4.0)	3 (4.5)	3 (3.5)	
Very low birth weight (< 2000 g)	3 (2.0)	0 (0.0)	3 (3.5)	
Average birth length (cm)^c^	50.9 ± 1.4	51.1 ± 1.3	50.7 ± 1.5	0.1295

We compiled the data for 153 people for whom we were able to obtain postnatal data. There were 67 males (43.8%) and 86 females (56.2%). Three pairs of twins (3/156: 1.9%) were born, but they were not included in this table

*GA* gestational age, *BW* birth weight

^a^One participant who delivered a male newborn did not answer the questionnaire

^b^One participant in each sex group did not answer the questionnaire

^c^One participant did not answer the questionnaire

### Relationship between carrier status and birth outcomes

The relationship between the copy number of each bacterium and birth outcomes was analyzed in univariate (Additional file 1: Table S2) and multivariate analyses (Table 3). In the analysis using the copy number of total bacteria as the objective variable, birth weight showed a negative correlation with copy number, and birth length showed a positive correlation in the multivariate analysis (*p* = 0.0032, *p* = 0.0286). In the analysis using the copy number of *P. gingivalis* as the objective variable, the sex ratio at birth (male births per female births) was significantly higher in pregnant women with a high copy number (univariate: *p* = 0.0046, multivariate: *p* = 0.0268).

**Table 3:** Correlation between the copy number of each bacterium and birth outcomes by multivariate analysis

Bacterial species	Variables	Estimate	Std. Error	t value	*p* value
Total Bacteria	Intercept	0.0514	0.0881	0.583	0.5609
	Age of the mother	0.0619	0.0887	0.698	0.4865
	Birth weight	−0.3278	0.1089	−3.012	0.0032**
	Birth length	0.2268	0.1024	2.215	0.0286*
	Gestational age	0.1047	0.1199	0.873	0.3844
	Sex of the newborn	0.0933	0.0886	1.054	0.2942
*P. gingivalis*	Intercept	−0.0596	0.0602	−0.991	0.3237
	Age of the mother	0.1059	0.0606	1.747	0.0831
	Birth weight	−0.1051	0.0744	−1.412	0.1604
	Birth length	0.1037	0.0700	1.482	0.1409
	Gestational age	0.0122	0.0819	0.149	0.8816
	Sex of the newborn	0.1356	0.0605	2.241	0.0268*
*T. forsythia*	Intercept	−0.0235	0.0727	−0.324	0.7467
	Age of the mother	0.1832	0.0732	2.502	0.0136*
	Birth weight	−0.2096	0.0898	−2.333	0.0212*
	Birth length	0.1901	0.0845	2.250	0.0262*
	Gestational age	−0.0426	0.0989	−0.430	0.6679
	Sex of the newborn	0.1384	0.0731	1.893	0.0606
*T. denticola*	Intercept	0.0225	0.0822	0.274	0.7848
	Age of the mother	0.0774	0.0828	0.934	0.3522
	Birth weight	−0.2248	0.1016	−2.212	0.0288*
	Birth length	0.2862	0.0956	2.994	0.0033**
	Gestational age	−0.2143	0.1119	−1.914	0.0579
	Sex of the newborn	0.2404	0.0827	2.908	0.0043**
*P. intermedia*	Intercept	0.0252	0.0950	0.265	0.7910
	Age of the mother	−0.0576	0.0957	−0.602	0.5480
	Birth weight	−0.0802	0.1174	−0.683	0.4960
	Birth length	0.0071	0.1104	0.065	0.9480
	Gestational age	0.0412	0.1293	0.319	0.7510
	Sex of the newborn	0.1293	0.0955	1.354	0.1780

A generalized linear model for multivariate analysis was performed using neonatal data to analyze the relationship between the copy number of each bacterium and birth outcomes. * indicate p value < 0.05 and ** indicate p value < 0.01

In the multivariate analysis, maternal age and birth length showed a positive correlation with the copy number of *T. forsythia* (*p* = 0.0136, *p* = 0.0262, respectively). On the other hand, birth weight showed a negative correlation with the copy number of *T. forsythia* (*p* = 0.0212).

In the analysis regarding the bacterial mass of *T. denticola*, the univariate analysis showed a positive correlation with birth length and sex ratio at birth (*p* = 0.0416, *p* = 0.0003, respectively). In the multivariate analysis, birth weight was negatively correlated with bacterial mass (*p* = 0.0288), and birth length was positively correlated (*p* = 0.0033). In addition, a large amount of bacterial mass showed a significant positive correlation with the sex ratio at birth (*p* = 0.0043).

The bacterial mass of *P. intermedia* was not significantly correlated with any of the birth outcomes in either the univariate or multivariate analysis.

### Tooth brushing habits of the participants

In terms of the frequency of daily tooth brushing among the participants, 75.7% (n = 115) brushed only once a day (Table 4). In addition, 74.0% (n = 111) of the participants did not change their toothbrush for 6 months or more, and 6.5% (n = 10) did not use a toothbrush. A total of 86.3% (n = 132) of the participants used both a brush and toothpaste when brushing their teeth, 7.2% (n = 11) used only a toothbrush, and 5.2% (n = 8) cleaned their teeth with a stick or finger. In addition, 1.3% (n = 2) of the participants gargled, and 14.4% (n = 22) had a history of visiting a dental clinic.

**Table 4:** Oral care status during pregnancy

Frequency of daily tooth brushing^a^	
1 time	115 (75.7)
2 times	36 (23.7)
3 times	1 (0.7)
Frequency of brush replacement^b^	
3 months	39 (26.0)
6 months	21 (14.0)
1 year or more	80 (53.3)
No brush used	10 (6.7)
Oral cleaning method	
Brush and paste	132 (86.3)
Brush	11 (7.2)
Stick or finger	8 (5.2)
Gargle	2 (1.3)
Lifetime dental examination history	22 (14.4)

Data from 153 participants were compiled. All values represent n (%)

^a^Data on the frequency of daily tooth brushing are missing for 1 woman

^b^Data on the frequency of brush replacement are missing for 3 women

### Relationship between tooth brushing habits and carrier status

An analysis of the relationship between the frequency of tooth brushing and the copy number of each bacterium was conducted. The copy number of *P. gingivalis* was significantly lower in the group of pregnant women who brushed their teeth more than once a day than in the group of pregnant women who brushed only once per day (*p* = 0.0061) (Table 5). In addition, the copy numbers of *P. gingivalis* and *T. forsythia* were significantly lower in the group of pregnant women who changed their toothbrush at 3 months than in the group of pregnant women who did not change their toothbrush at 6 months or more (*p* = 0.0153, *p* = 0.0029, respectively). In addition, the pregnant women with a history of dental examination had significantly lower levels of all four species than those without a history of dental examination (*P. gingivalis*: *p* = 0.0032; *T. forsythia*: *p* = 0.0093; *T. denticola*: *p* = 0.0054; and *P. intermedia*: *p* = 0.0448). However, the tools for tooth brushing were not related to the copy number of any of the bacterial species.

**Table 5:** Comparison of the copy numbers of bacteria by oral cleaning habit

Bacterial species	Frequency of daily tooth brushing		*p* value
	2 times or more	1 time	
Total bacteria	9.83E6 ± 1.10E7	1.11E7 ± 1.17E7	0.5463
*P. gingivalis*	5.56E4 ± 8.47E4	1.72E5 ± 4.22E5	0.0061
*T. forsythia*	8.28E3 ± 1.47E4	1.27E4 ± 1.82E4	0.1353
*T. denticola*	1.93E5 ± 3.64E5	2.28E5 ± 3.54E5	0.6103
*P. intermedia*	1.40E4 ± 4.10E4	1.03E4 ± 1.69E4	0.5987

Bacterial species	Frequency of brush replacement		*p* value
	3 months	6 months or more	
Total bacteria	9.85E6 ± 1.14E7	1.11E7 ± 1.15E7	0.5588
*P. gingivalis*	6.48E4 ± 1.05E5	1.70E5 ± 4.23E5	0.0153
*T. forsythia*	6.80E3 ± 7.63E3	1.35E4 ± 1.95E4	0.0029
*T. denticola*	1.82E5 ± 2.98E5	2.32E5 ± 3.72E5	0.4011
*P. intermedia*	1.45E4 ± 4.01E4	1.00E4 ± 1.68E4	0.5010

Bacterial species	Brush tool		*p* value
	Brush and paste	Other	
Total bacteria	1.06E7 ± 1.13E7	1.17E7 ± 1.30E7	0.7186
*P. gingivalis*	9.45E4 ± 2.13E5	4.51E5 ± 7.97E5	0.0545
*T. forsythia*	1.09E4 ± 1.58E4	1.70E4 ± 2.55E4	0.2996
*T. denticola*	2.09E5 ± 3.45E5	2.88E5 ± 4.11E5	0.4093
*P. intermedia*	1.16E4 ± 2.63E4	8.75E3 ± 1.13E4	0.4063

Bacterial species	Lifetime dental examination history		*p* value
	Experience	Never	
Total bacteria	8.95E6 ± 9.19E6	1.11E7 ± 1.19E7	0.3424
*P. gingivalis*	4.20E4 ± 8.90E4	1.61E5 ± 3.97E5	0.0032
*T. forsythia*	6.38E3 ± 8.00E3	1.27E4 ± 1.85E4	0.0093
*T. denticola*	1.08E5 ± 1.48E5	2.38E5 ± 3.76E5	0.0054
*P. intermedia*	6.14E3 ± 8.15E3	1.20E4 ± 2.65E4	0.0448

Data from 153 participants were compiled. In this table, the copy number of each bacterium is displayed as the average ± SD

^a^Data on the frequency of daily tooth brushing is missing for 1 person

^b^Data on the frequency of brush replacement are missing for 3 people

## Discussion

### Age of pregnant women and fertility rate in Rwanda

A previous epidemiological survey targeting 7525 pregnant women in 30 districts in Rwanda found that 17.0% of pregnant women were over 35 years old [13], and in a survey of 2,150 pregnant women in four districts, 18.7% were aged 35 years and over [14]. On the other hand, in a survey targeting pregnant women in the capital Kigali, only 12.5% of the participants were over the age of 35 years [15]. Older pregnant women, including those in their 40 s, were reported particularly in the Mibilizi Hospital zone, which was surveyed in this study. These findings suggest that the average age of pregnant women is higher in rural areas than in urban areas.

In Africa, the total fertility rate of women aged 15–49 years is known to be higher in rural areas than in urban areas [16]. The total fertility rate in Rwanda peaked at 8.46 in 1979 and declined to 3.99 in 2019 [17], and a national survey in 2015 reported that the total fertility rate was 3.6 in the capital Kigali but 4.6 in the western province where the hospital is located [18]. In rural areas, such as the zone where Mibilizi Hospital is located, many women become pregnant even after the age of 35 years, which is considered to be a factor in the high fertility rate in rural areas.

### Relationship between carrier status and birth outcomes

The prevalence of periodontitis increases with age [19]. In this study, the copy number of *T. forsythia*, which is the causative agent of severe periodontal disease, tended to increase as the age of the pregnant women increased, which is consistent with previous reports. Although the other bacterial species detected in this study did not correlate with the age of the pregnant women, our findings suggest that the bacterial species, whose mass is affected by the aging of the host, may differ depending on the lifestyle, eating habits or tooth brushing habits of the women.

It has been reported that periodontal disease among pregnant women promotes an inflammatory reaction and increases the risk of low birth weight [20]. In this study, we further analyzed the relationship between the amount of bacteria and birth outcomes and tried to identify which bacterial species particularly affected birth outcomes. Regarding the development of the fetus, the copy numbers of total bacteria and *T. forsythia* and *T. denticola* were correlated with birth weight in this study. Previous studies have also reported that a large copy number of *T. denticola* in the oral cavity increases the risk of preterm low birth weight in China [5], so it is reasonable to assume that *T. denticola* generally influences fetal development regardless of the country.

Our findings also suggested that there may be weak correlation between the copy number of *T. denticola* and gestational age even though *p* value was not under 0.05. It’s possible that the high copy number of *T. denticola* affect to the reduction in both gestational age and birth weight in this area.

Previous studies have suggested that infections in pregnant women, especially oral, intrauterine, amniotic fluid, urinary tract, vaginal infections, and pneumonia, are associated with preterm birth [21, 22]. In particular, it has been suggested that periodontal bacteria induce preterm birth through increased levels of inflammatory cytokines and PGE2 [23]. However, in reality, many studies have investigated the relationship between the progression of periodontal disease and pregnancy outcomes. In these studies, periodontal status was assessed by measuring the bleeding tendency from the gums, the depth of the periodontal pocket and so on. In addition, some reports stated that there was no correlation between the prevalence of preterm low birth weight and the severity of periodontal disease [24]. In this study, we focused on periodontal bacteria, which are considered to be the cause of periodontal disease and induce pregnancy abnormalities, and tried to clarify the relationship between the amount of bacteria in the oral cavity of pregnant women and pregnancy outcomes in Rwanda. It was reported that the detection rate of *T. denticola* from the placenta was higher in pregnant women with threatened preterm birth than in normal pregnant women [25]. Although only a weak correlation was found in this study, an increase in the number of *T. denticola* in the oral cavity may be a risk factor for preterm birth.

The sex ratio at birth was significantly higher in the pregnant women with high copy numbers of *P. gingivalis* and *T. denticola*. Previous studies have reported that the sex of the fetus may affect the maternal immune response. For example, when the serum of women who were pregnant with a female fetus was stimulated by lipopolysaccharide, they produced higher interleukin-6 (IL-6) and interleukin-1β (IL-1β) levels than women who were pregnant with a male fetus [26]. There is also a report that severe acute respiratory syndrome coronavirus 2 (SARS-CoV-2)-specific antibody titers are lower in pregnant women with coronavirus disease 2019 (COVID-19) when the fetus was a male [27]. Thus, if the fetus is a male, the immune response of the mother may be suppressed and may lead to increased cytokine levels and antibody titers. Therefore, the immune response in pregnant women with male fetuses might be suppressed, and it might be reasonable to hypothesize that the growth of periodontal bacteria is promoted in the oral cavity of these women.

Transglutaminase 2 (TG2), a protein cross-linking enzyme, is thought to be associated with the activation of NF-κB, which is a regulator of inflammation. In fact, human periodontal ligament cells (HPDL cells) collected from patients with chronic periodontitis had higher levels of inflammatory cytokines, including IL-6, TNF-α, and HMGB-1, than HDPL cells collected from healthy individuals. In addition, the TG2 mRNA levels were also higher. In other words, TG2 may be involved in the early stages of the inflammatory response that occurs in periodontal disease. Therefore, measuring TG2 levels in the blood is also useful for monitoring inflammation during pregnancy, and in the future, simultaneous observation of the carrying status of periodontal bacteria and blood TG2 levels during pregnancy may clarify the relationship of TG2 with all pregnancy outcomes [28]. It has also been reported that celecoxib is more effective than ibuprofen and placebos in terms of the incidence and severity of postoperative pain caused by the surgical removal of teeth. Pain intensity was assessed by a visual analog scale (VAS) in this study, and the anti-inflammatory effects of celecoxib may also be effective in preventing periodontitis [29]. Further verification is required to see if celecoxib also has a preventive effect against excessive inflammatory reactions in pregnant women.

### Oral cleaning habits among pregnant women

Tooth brushing at least twice a day is recommended to maintain oral hygiene [30]. A survey of pregnant women aged 15–43 years in rural Zambia found that 38.5% of the participants brushed their teeth more than once a day [31]. As a result of the current study, we speculate that pregnant women, especially in the Mibilizi Hospital zone, may not brush their teeth sufficiently to maintain oral hygiene. Furthermore, in a survey of adults living in the capital of Kigali, the percentage of participants who brushed their teeth more than once a day was only 27.2% [32]. This suggests that tooth brushing guidance and education may be required in both urban and rural areas of Rwanda. A study has shown that the frequency of tooth brushing decreases during pregnancy [33]; therefore, oral cleaning guidance for pregnant women is even more important.

The frequency of toothbrush replacement also affects oral hygiene. Worn toothbrushes lose their ability to remove plaque; thus, it is recommended that toothbrushes be replaced when the bristles are worn or every 3 to 4 months of use at least [34]. More than half of the pregnant women in the Mibilizi Hospital zone did not replace their toothbrush for more than a year. Considering that there are few opportunities to replace toothbrushes in rural than in urban areas, brushing teeth at least twice a day is considered to be particularly important in rural areas.

In addition to oral cleaning habits, restricted access to dental clinics is also a cause of deterioration of the oral environment. In a study conducted in Nigeria, only 12.5% of pregnant women had a history of access to a dental clinic [35], similar to the participants of the current study. There is a report that the presence of untreated dental caries and tartar is higher in rural areas than in urban areas [36], and the morbidity and severity associated with periodontal disease may also be related to accessibility to dental clinics.

Tooth brushing habits generally tend to deteriorate in the elderly generation [37, 38]. In the subjects in this study, the frequency of toothbrush replacement decreased and the proportion of those who brushed their teeth using inappropriate appliances increased with age in pregnant women (Additional file 1: Table S3); interventions need to be developed with regard to age for pregnant woman.

### Effect of oral cleaning habits on carrier status

Among the participants in this study, the copy number of *P. gingivalis* was significantly higher in the pregnant women who brushed their teeth less frequently. Less frequent brushing has been reported to increase the prevalence of severe periodontal disease [39] and increase the number of positive bacterial species in the periodontal pocket [40]. These results suggest that poor tooth brushing habits of the pregnant women in the Mibilizi Hospital zone may increase the risk of periodontal disease. In addition, since the copy numbers of *P. gingivalis* and *T. forsythia* were high in the pregnant women who infrequently changed their toothbrushes, it is necessary to provide health guidance and material support so that toothbrushes are changed every 3 months of use.

Insufficient tooth brushing habits increase periodontal bacteria and promote oral inflammation. The nucleotide-binding oligomerization domain-like receptor family pyrin domain-containing 3 (NLRP3) is one of the constituents of the inflammasome, a protein complex involved in inflammatory response mechanisms. When NLRP3 knockout mice and wild-type mice were exposed to *P. gingivalis*, alveolar bone resorption was not enhanced in the knockout mice, suggesting that NLRP3 is strongly involved in *P. gingivalis*-induced bone metabolism and bone resorption, that is, alveolar bone dissolution [41]. Metformin, an oral antidiabetic drug, exhibits anti-inflammatory effects by reducing the activity of inflammasomes containing NLRP3, suggesting that anti-inflammatory drugs may be useful in the prevention and treatment of periodontal disease [42]. In addition, it has been reported that many drugs, such as allopurinol and nicotinamide riboside, inhibit intracellular signaling, and pralnacasan and emricasan inhibit caspase-1, and that many drugs act on various stages of the inflammatory reaction to exhibit anti-inflammatory responses [43]. However, it is not easy to introduce these anti-inflammatory drugs in rural Africa and distribute them in a short period. On the other hand, chlorhexidine is a skin antiseptic that is used in a wide range of applications because it binds strongly to proteins in the skin and mucous membranes, which makes it easier to maintain its antibacterial effect. It is used not only for the skin but also as an ingredient in toothpaste and mouthwash [44]. Various studies have reported the efficacy of chlorhexidine in perinatal care, including the efficacy of vaginal swabs and neonatal skin cleansing with chlorhexidine in Malawi and the efficacy of umbilical cord cleansing in Nepal [45]. In dentistry, it has been shown that the combination of scaling and root planning (SRP), which removes plaque and polishes the tooth surface, with chlorhexidine irrigation improves periodontitis more than SRP alone [46]. In addition, a study suggested that routine chlorhexidine mouthwashes may reduce the risk of premature birth [47]. This study revealed that inadequate tooth brushing habits were associated with an increase in periodontal bacteria in the target population and that high amounts of periodontal bacteria correlated with lower birth weight. Therefore, chlorhexidine is effective in preventing preterm low birth weight, especially in rural Africa, where dental care resources are limited.

Our findings also indicate that it is important to visit a dental clinic to prevent periodontal disease and preterm low birth weight because the copy numbers of all four bacterial species tended to be low in the group of pregnant women who had visited a dental clinic. As shown in Additional file 1: Supplementary figure 1, the number of dentists per population in Rwanda is small compared with other less developed countries or low- and middle-income countries, such as Peru and Nepal. The number of dentists per 100,000 population is less than 10 in many countries, and Rwanda, which has 1.9 dentists per 100,000 population, can be said to have a serious shortage of dentists [48]. If both access to dental clinics in rural areas and the oral cleaning habits of pregnant women are improved, birth outcomes may gradually improve. Furthermore, it is necessary not only to improve the antenatal care visit rate but also to incorporate dental examination as part of antenatal care.

In this study, for the first time, we simultaneously analyzed the relationship between the amount of periodontal bacteria in the oral cavity, tooth brushing habits, and pregnancy outcomes in pregnant women in Africa. Other preterm birth risk factors were also investigated. It was previously revealed that the risk of preterm low birth weight was increased in those with low lipid and vitamin E intake during pregnancy [49], malaria infection inhibiting angiogenesis and nutrient transport in the placenta [50], urinary schistosomiasis [51] and luxury goods, such as tobacco, consumption [52]. Therefore, it is necessary to clarify in more detail the effects of periodontal bacteria on birth outcomes, taking into consideration the lifestyle and socioeconomic factors of local pregnant women and infectious diseases other than periodontal disease.

## Conclusion

This study showed that carrying a high copy number of periodontal bacteria during pregnancy may be associated with a risk of low birth weight in rural Rwanda. In addition, inadequate oral cleaning habits are associated with an increase in the copy number of bacteria in the oral cavity, and the oral condition during pregnancy may affect not only birth weight, which have been the focus of previous studies, but also other birth outcomes, including the sex of the fetus. Therefore, especially in rural Africa, where medical resources are limited, tooth brushing guidance, which includes the recommendation for using chlorhexidine mouthwash and improved access to dental clinics, is important to prevent periodontal disease among pregnant women and women who may become pregnant and to reduce the burden of premature birth and low birth weight.

## Supplementary Information

**Additional file 1**. Sequence data of primers and probes used to detect periodontal bacteria, results of univariate analysis not shown in the text, relationship between age group of pregnant women and toothbrushing habits, and figure indicating the number of dentists per population by countries.

**Additional file 2**. Raw data of the number of bacteria calculated from the amount of DNA detected by real-time PCR.

## Data Availability

The datasets generated and/or analyzed during the current study are not publicly available but are available from the corresponding author on reasonable request.

## Abbreviations

BW: Birth Weight
COVID-19: Coronavirus Disease 2019
GA: Gestational Age
HMGB-1: High Mobility Group Box-1
HPDL cell: Human Periodontal Ligament cell
IL-1β: Interleukin-1β
IL-6: Interleukin-6
NF-κB: Nuclear Factor-kappa B
NLRP3: Nucleotide-binding oligomerization domain-like receptor family pyrin domain-containing 3
PGE2: Prostaglandin E2
SARS-CoV-2: Severe Acute Respiratory Syndrome Coronavirus 2
TG2: Transglutaminase 2

## References

[1] Huck O, Tenenbaum H, Davideau JL (2011). Relationship between periodontal diseases and preterm birth: recent epidemiological and biological data. J Pregnancy.

[2] Saini R, Saini S, Saini SR (2011). Periodontitis: a risk for delivery of premature labor and low birth weight infants. J Nat Sci Biol Med.

[3] Dörtbudak O, Eberhardt R, Ulm M, Persson GR (2005). Periodontitis, a marker of risk in pregnancy for preterm birth. J Clin Periodontol.

[4] Kim E-H, Kim S, Kim H-J, Jeong H-o, Lee J, Jang J, et al. Prediction of chronic periodontitis severity using machine learning models based on salivary bacterial copy number. Front Cell Infect Microbiol. 2020;10. 10.3389/fcimb.2020.571515 PMC7701273 33304856

[5] Ye C, Xia Z, Tang J, Khemwong T, Kapila Y, Kuraji R (2020). Unculturable and culturable periodontal-related bacteria are associated with periodontal inflammation during pregnancy and with preterm low birth weight delivery. Sci Rep.

[6] Periodontology EFo. Why it matters. https://www.efp.org/gum-disease-general-health/oral-health-pregnancy/overview/why-it-matters/. 2021;Accessed on 5 Nov 2021.

[7] Prevention CfDCa. Pregnancy and Oral Health. https://www.cdc.gov/oralhealth/publications/features/pregnancy-and-oral-health.html. 2019;Accessed on 5 Nov 2021.

[8] Martellacci L, Quaranta G, Patini R, Isola G, Gallenzi P, Masucci L. A literature review of metagenomics and culturomics of the peri-implant microbiome: current evidence and future perspectives. Materials (Basel). 2019;12(18). 10.3390/ma12183010 PMC6766346 31533226

[9] Mason MR, Nagaraja HN, Camerlengo T, Joshi V, Kumar PS (2013). Deep sequencing identifies ethnicity-specific bacterial signatures in the oral microbiome. PLoS ONE.

[10] Authority WSHC. Prenatal screening guide. https://www.hca.wa.gov/billers-providers-partners/programs-and-services/first-steps-maternity-and-infant-care. 2014; Accessed on 1 Sept 2018.

[11] Martin FE, Nadkarni MA, Jacques NA, Hunter N (2002). Quantitative microbiological study of human carious dentine by culture and real-time PCR: association of anaerobes with histopathological changes in chronic pulpitis. J Clin Microbiol.

[12] Kuboniwa M, Amano A, Kimura KR, Sekine S, Kato S, Yamamoto Y (2004). Quantitative detection of periodontal pathogens using real-time polymerase chain reaction with TaqMan probes. Oral Microbiol Immunol.

[13] Sayinzoga F, Lundeen T, Musange SF, Butrick E, Nzeyimana D, Murindahabi N (2021). Assessing the impact of group antenatal care on gestational length in Rwanda: a cluster-randomized trial. PLoS ONE.

[14] Miller P, Afulani PA, Musange S, Sayingoza F, Walker D (2021). Person-centered antenatal care and associated factors in Rwanda: a secondary analysis of program data. BMC Pregnancy Childbirth.

[15] Nsereko E, Uwase A, Mukabutera A, Muvunyi CM, Rulisa S, Ntirushwa D (2020). Maternal genitourinary infections and poor nutritional status increase risk of preterm birth in Gasabo District, Rwanda: a prospective, longitudinal, cohort study. BMC Pregnancy Childbirth.

[16] Lesthaeghe R. The Fertility Transition in Sub Sahara Africa into the 21st Century ( full version 24 oct 2013). unpublished. 2013.

[17] Bank TW. Fertility rate, total (births per woman) - Rwanda. https://data.worldbank.org/indicator/SPDYNTFRTIN?locations=RW. 2018; Accessed on 3 Sept 2021.

[18] National Institute of Statistics of Rwanda MoH, and ICF , International. Rwanda 2014–15 Demographic and Health Survey Key Findings. https://www.dhsprogram.com/pubs/pdf/SR229/SR229pdf. 2016; Accessed on 20 May 2022.

[19] Nazir M, Al-Ansari A, Al-Khalifa K, Alhareky M, Gaffar B, Almas K (2020). Global prevalence of periodontal disease and lack of its surveillance. ScientificWorldJournal.

[20] Teshome A, Yitayeh A (2016). Relationship between periodontal disease and preterm low birth weight: systematic review. Pan Afr Med J.

[21] Gonçalves LF, Chaiworapongsa T, Romero R (2002). Intrauterine infection and prematurity. Ment Retard Dev Disabil Res Rev.

[22] Walia M, Saini N (2015). Relationship between periodontal diseases and preterm birth: recent epidemiological and biological data. Int J Appl Basic Med Res.

[23] Latorre Uriza C, Velosa-Porras J, Roa NS, Quiñones Lara SM, Silva J, Ruiz AJ (2018). Periodontal disease, inflammatory cytokines, and PGE(2) in pregnant patients at risk of preterm delivery: a pilot study. Infect Dis Obstet Gynecol.

[24] Daalderop LA, Wieland BV, Tomsin K, Reyes L, Kramer BW, Vanterpool SF (2018). Periodontal disease and pregnancy outcomes: overview of systematic reviews. JDR Clin Trans Res.

[25] Ye C, Katagiri S, Miyasaka N, Kobayashi H, Khemwong T, Nagasawa T (2020). The periodontopathic bacteria in placenta, saliva and subgingival plaque of threatened preterm labor and preterm low birth weight cases: a longitudinal study in Japanese pregnant women. Clin Oral Invest.

[26] Mitchell AM, Palettas M, Christian LM (2017). Fetal sex is associated with maternal stimulated cytokine production, but not serum cytokine levels, in human pregnancy. Brain Behav Immun.

[27] Bordt EA, Shook LL, Atyeo C, Pullen KM, De Guzman RM, Meinsohn MC, et al. Maternal SARS-CoV-2 infection elicits sexually dimorphic placental immune responses. Sci Transl Med. 2021;13(617):eabi7428. 10.1126/scitranslmed.abi7428 PMC8784281 34664987

[28] Matarese G, Currò M, Isola G, Caccamo D, Vecchio M, Giunta ML (2015). Transglutaminase 2 up-regulation is associated with RANKL/OPG pathway in cultured HPDL cells and THP-1-differentiated macrophages. Amino Acids.

[29] Isola G, Matarese M, Ramaglia L, Cicciù M, Matarese G (2019). Evaluation of the efficacy of celecoxib and ibuprofen on postoperative pain, swelling, and mouth opening after surgical removal of impacted third molars: a randomized, controlled clinical trial. Int J Oral Maxillofac Surg.

[30] Fernandez de Grado G, Ehlinger V, Godeau E, Arnaud C, Nabet C, Benkirane-Jessel N, et al. Changes in tooth brushing frequency and its associated factors from 2006 to 2014 among French adolescents: results from three repeated cross sectional HBSC studies. PLOS ONE. 2021;16(3):e0249129. 10.1371/journal.pone.0249129 PMC8007017 33780479

[31] Kabali TM, Mumghamba EG (2018). Knowledge of periodontal diseases, oral hygiene practices, and self-reported periodontal problems among pregnant women and postnatal mothers attending reproductive and Child Health Clinics in Rural Zambia. Int J Dentistry.

[32] Janvière Mutamuliza FR, Stephen Rulisa and Joseph Ntaganira. Prevalence and associated risk factors of periodontal disease among adults attending Dental Department in Rwanda Military Hospital (Rwanda): a cross sectional study. Dentistry. 2015;2(4).

[33] Martins R, Azevedo J, Dourado C, Ribeiro C, Alves C, Thomaz E (2014). Oral health behaviors and dental treatment during pregnancy: a cross-sectional study nested in a cohort in Northeast Brazil. Pesquisa Brasileira em Odontopediatria e Clínica Integrada.

[34] Ss R (2015). An investigation into toothbrush wear related to months of use among university students. Can J Dental Hygiene.

[35] Ifesanya JU, Ifesanya AO, Asuzu MC, Oke GA (2010). Determinants of good oral hygiene among pregnant women in ibadan. South-Western Nigeria Ann Ib Postgrad Med.

[36] Hackley DM, Jain S, Pagni SE, Finkelman M, Ntaganira J, Morgan JP (2021). Oral health conditions and correlates: a National Oral Health Survey of Rwanda. Glob Health Action.

[37] Islas-Granillo H, Casanova-Rosado JF, de la Rosa-Santillana R, Casanova-Rosado AJ, Islas-Zarazúa R, Márquez-Corona ML (2020). Self-reported oral hygiene practices with emphasis on frequency of tooth brushing: A cross-sectional study of Mexican older adults aged 60 years or above. Medicine (Baltimore).

[38] Melo P, Marques S, Silva OM (2017). Portuguese self-reported oral-hygiene habits and oral status. Int Dent J.

[39] Zimmermann H, Zimmermann N, Hagenfeld D, Veile A, Kim TS, Becher H (2015). Is frequency of tooth brushing a risk factor for periodontitis? A systematic review and meta-analysis. Community Dent Oral Epidemiol.

[40] Pyysalo MJ, Mishra PP, Sundström K, Lehtimäki T, Karhunen PJ, Pessi T. Increased tooth brushing frequency is associated with reduced gingival pocket bacterial diversity in patients with intracranial aneurysms. PeerJ. 2019 2019; 7:[e6316 p.]. 10.7717/peerj.6316 PMC6348950 30701137

[41] Yamaguchi Y, Kurita-Ochiai T, Kobayashi R, Suzuki T, Ando T (2017). Regulation of the NLRP3 inflammasome in Porphyromonas gingivalis-accelerated periodontal disease. Inflamm Res.

[42] Tan Y, Chen J, Jiang Y, Chen X, Li J, Chen B (2020). The anti-periodontitis action of metformin via targeting NLRP3 inflammasome. Arch Oral Biol.

[43] Rana A, Kaur A, Faraz F, Tandon S (2021). Targeting inflammasomes: A possible therapeutic approach for periodontal disease (Review). World Acad Sci J.

[44] Lim KS, Kam PC (2008). Chlorhexidine–pharmacology and clinical applications. Anaesth Intensive Care.

[45] McClure EM, Goldenberg RL, Brandes N, Darmstadt GL, Wright LL, Armbruster D (2007). The use of chlorhexidine to reduce maternal and neonatal mortality and morbidity in low-resource settings. Int J Gynaecol Obstet.

[46] Verma A, Sanghi S, Grover D, Aggarwal S, Gupta R, Pandit N (2012). Effect of insertion of xanthan-based chlorhexidine gel in the maintenance phase following the treatment of chronic periodontitis. J Indian Soc Periodontol.

[47] Boutin A, Demers S, Roberge S, Roy-Morency A, Chandad F, Bujold E (2013). Treatment of periodontal disease and prevention of preterm birth: systematic review and meta-analysis. Am J Perinatol.

[48] Organization WH. Dentistry personnel. https://apps.who.int/gho/data/nodemainHWF2. 2021;Accessed on 4 Sept 2021.

[49] Zhang Y, Zhou H, Perkins A, Wang Y, Sun J. Maternal dietary nutrient intake and its association with preterm birth: a case-control study in Beijing, China. Nutrients. 2017;9(3).

[50] Chua CLL, Hasang W, Rogerson SJ, Teo A (2021). Poor birth outcomes in malaria in pregnancy: recent insights into mechanisms and prevention approaches. Front Immunol.

[51] Mombo-Ngoma G, Honkpehedji J, Basra A, Mackanga JR, Zoleko RM, Zinsou J (2017). Urogenital schistosomiasis during pregnancy is associated with low birth weight delivery: analysis of a prospective cohort of pregnant women and their offspring in Gabon. Int J Parasitol.

[52] Salihu HM, Wilson RE (2007). Epidemiology of prenatal smoking and perinatal outcomes. Early Hum Dev.

