# Supplementary material for: Oral cleaning habits and the copy number of periodontal bacteria in pregnant women and its correlation with birth outcomes: an epidemiological study in Mibilizi, Rwanda
Source: BMC Oral Health. 2022 Sep 26;22:428. doi: 10.1186/s12903-022-02443-4 (PMC9512986; doi:10.1186/s12903-022-02443-4)
Supplement: Supplementary file 1 — Additional file 1. Sequence data of primers and probes used to detect periodontal bacteria, results of univariate analysis not shown in the text, relationship between age group of pregnant women and toothbrushing habits, and figure indicating the number of dentists per population by countries. [file 12903_2022_2443_MOESM1_ESM.pptx]

## Slide 1
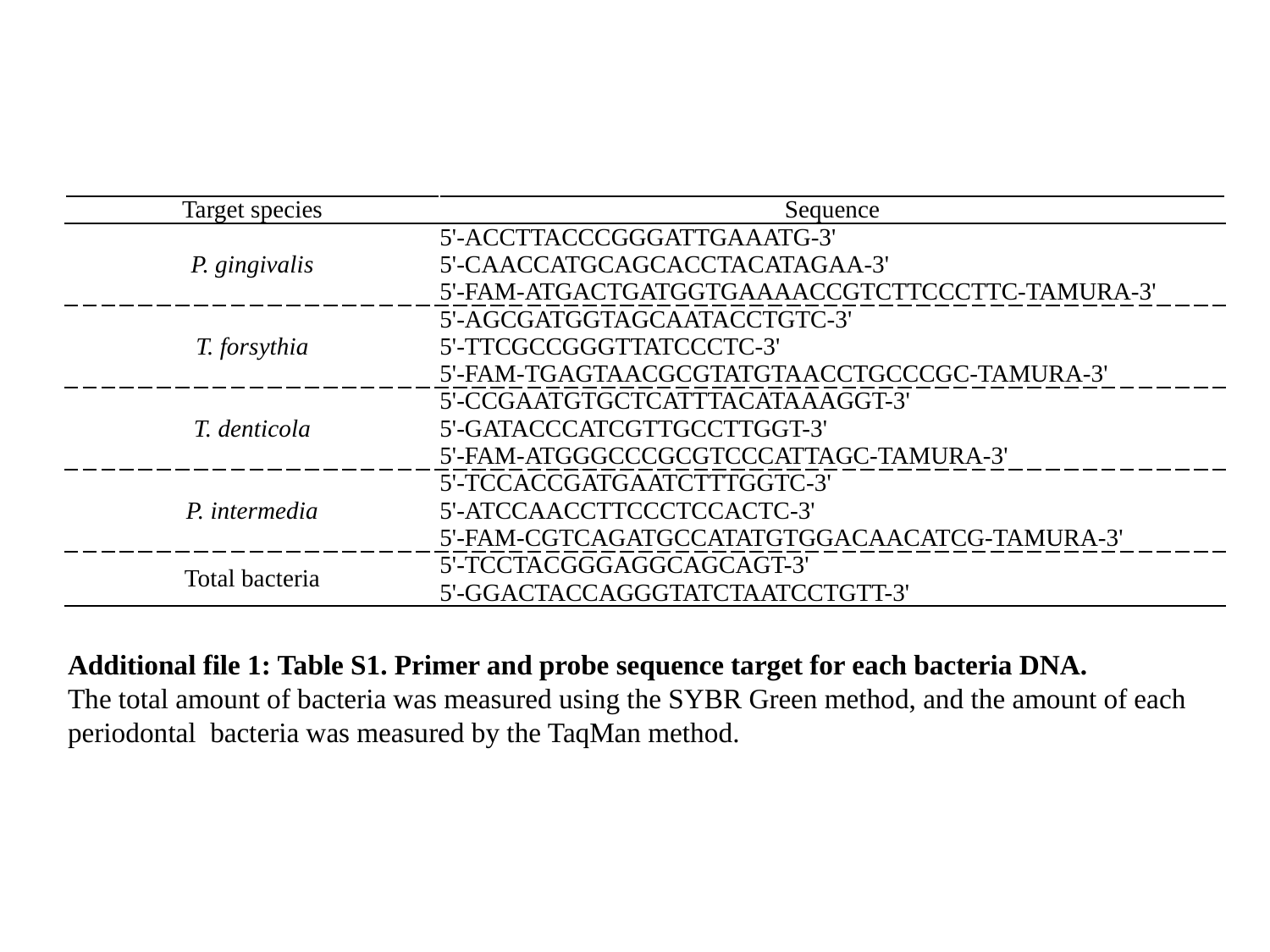

| Target species | Sequence |
| --- | --- |
| P. gingivalis | 5'-ACCTTACCCGGGATTGAAATG-3' |
| | 5'-CAACCATGCAGCACCTACATAGAA-3' |
| | 5'-FAM-ATGACTGATGGTGAAAACCGTCTTCCCTTC-TAMURA-3' |
| T. forsythia | 5'-AGCGATGGTAGCAATACCTGTC-3' |
| | 5'-TTCGCCGGGTTATCCCTC-3' |
| | 5'-FAM-TGAGTAACGCGTATGTAACCTGCCCGC-TAMURA-3' |
| T. denticola | 5'-CCGAATGTGCTCATTTACATAAAGGT-3' |
| | 5'-GATACCCATCGTTGCCTTGGT-3' |
| | 5'-FAM-ATGGGCCCGCGTCCCATTAGC-TAMURA-3' |
| P. intermedia | 5'-TCCACCGATGAATCTTTGGTC-3' |
| | 5'-ATCCAACCTTCCCTCCACTC-3' |
| | 5'-FAM-CGTCAGATGCCATATGTGGACAACATCG-TAMURA-3' |
| Total bacteria | 5'-TCCTACGGGAGGCAGCAGT-3' |
| | 5'-GGACTACCAGGGTATCTAATCCTGTT-3' |
Additional file 1: Table S1. Primer and probe sequence target for each bacteria DNA.
The total amount of bacteria was measured using the SYBR Green method, and the amount of each periodontal bacteria was measured by the TaqMan method.

## Slide 2
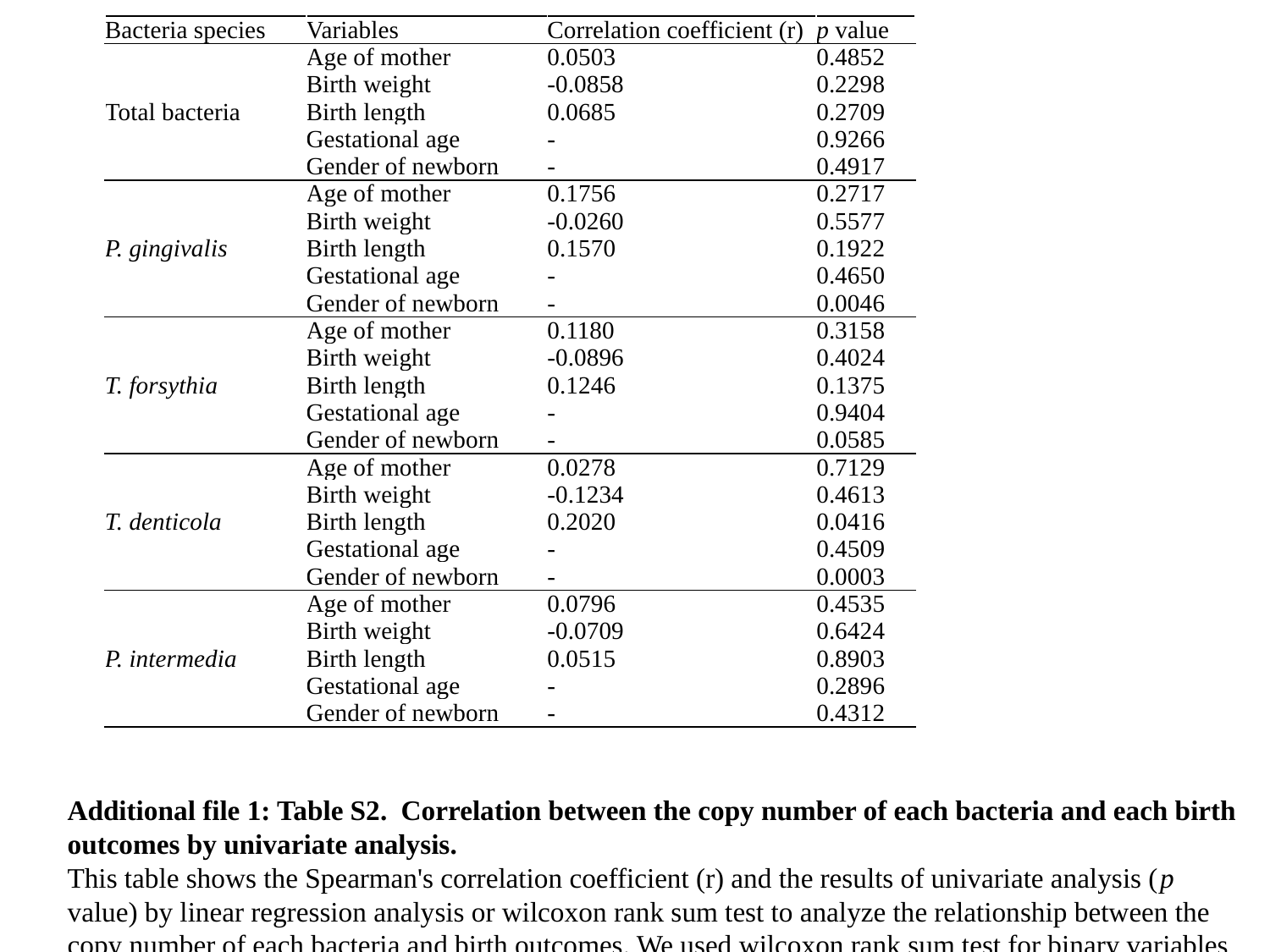

| Bacteria species | Variables | Correlation coefficient (r) | p value |
| --- | --- | --- | --- |
| Total bacteria | Age of mother | 0.0503 | 0.4852 |
| | Birth weight | -0.0858 | 0.2298 |
| | Birth length | 0.0685 | 0.2709 |
| | Gestational age | - | 0.9266 |
| | Gender of newborn | - | 0.4917 |
| P. gingivalis | Age of mother | 0.1756 | 0.2717 |
| | Birth weight | -0.0260 | 0.5577 |
| | Birth length | 0.1570 | 0.1922 |
| | Gestational age | - | 0.4650 |
| | Gender of newborn | - | 0.0046 |
| T. forsythia | Age of mother | 0.1180 | 0.3158 |
| | Birth weight | -0.0896 | 0.4024 |
| | Birth length | 0.1246 | 0.1375 |
| | Gestational age | - | 0.9404 |
| | Gender of newborn | - | 0.0585 |
| T. denticola | Age of mother | 0.0278 | 0.7129 |
| | Birth weight | -0.1234 | 0.4613 |
| | Birth length | 0.2020 | 0.0416 |
| | Gestational age | - | 0.4509 |
| | Gender of newborn | - | 0.0003 |
| P. intermedia | Age of mother | 0.0796 | 0.4535 |
| | Birth weight | -0.0709 | 0.6424 |
| | Birth length | 0.0515 | 0.8903 |
| | Gestational age | - | 0.2896 |
| | Gender of newborn | - | 0.4312 |
Additional file 1: Table S2. Correlation between the copy number of each bacteria and each birth outcomes by univariate analysis.
This table shows the Spearman's correlation coefficient (r) and the results of univariate analysis (p value) by linear regression analysis or wilcoxon rank sum test to analyze the relationship between the copy number of each bacteria and birth outcomes. We used wilcoxon rank sum test for binary variables in Gestational age and Gender of newborn.

## Slide 3
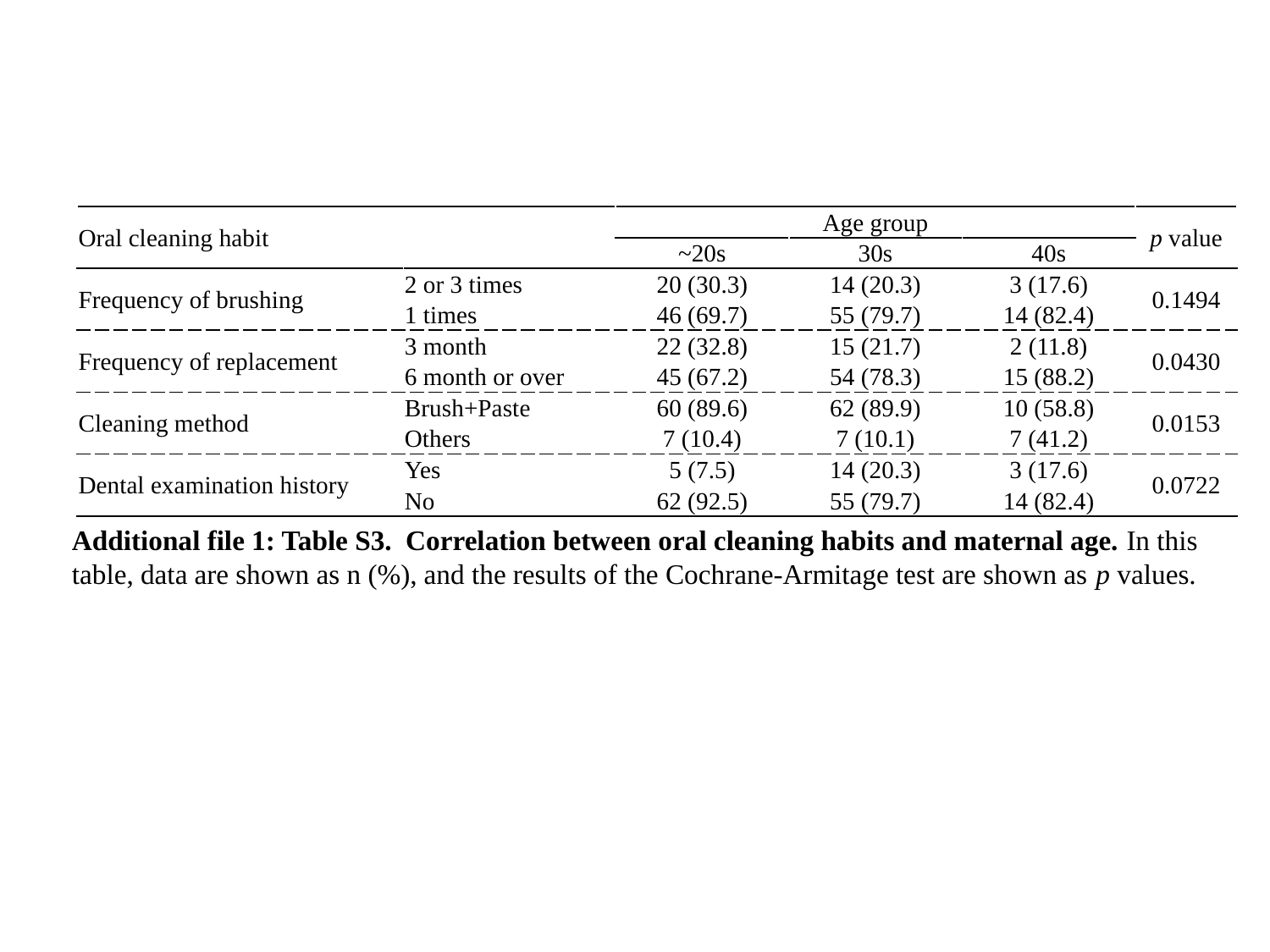

| Oral cleaning habit | | Age group | | | p value |
| --- | --- | --- | --- | --- | --- |
| | | ~20s | 30s | 40s | |
| Frequency of brushing | 2 or 3 times | 20 (30.3) | 14 (20.3) | 3 (17.6) | 0.1494 |
| | 1 times | 46 (69.7) | 55 (79.7) | 14 (82.4) | |
| Frequency of replacement | 3 month | 22 (32.8) | 15 (21.7) | 2 (11.8) | 0.0430 |
| | 6 month or over | 45 (67.2) | 54 (78.3) | 15 (88.2) | |
| Cleaning method | Brush+Paste | 60 (89.6) | 62 (89.9) | 10 (58.8) | 0.0153 |
| | Others | 7 (10.4) | 7 (10.1) | 7 (41.2) | |
| Dental examination history | Yes | 5 (7.5) | 14 (20.3) | 3 (17.6) | 0.0722 |
| | No | 62 (92.5) | 55 (79.7) | 14 (82.4) | |
Additional file 1: Table S3. Correlation between oral cleaning habits and maternal age. In this table, data are shown as n (%), and the results of the Cochrane-Armitage test are shown as p values.

## Slide 4
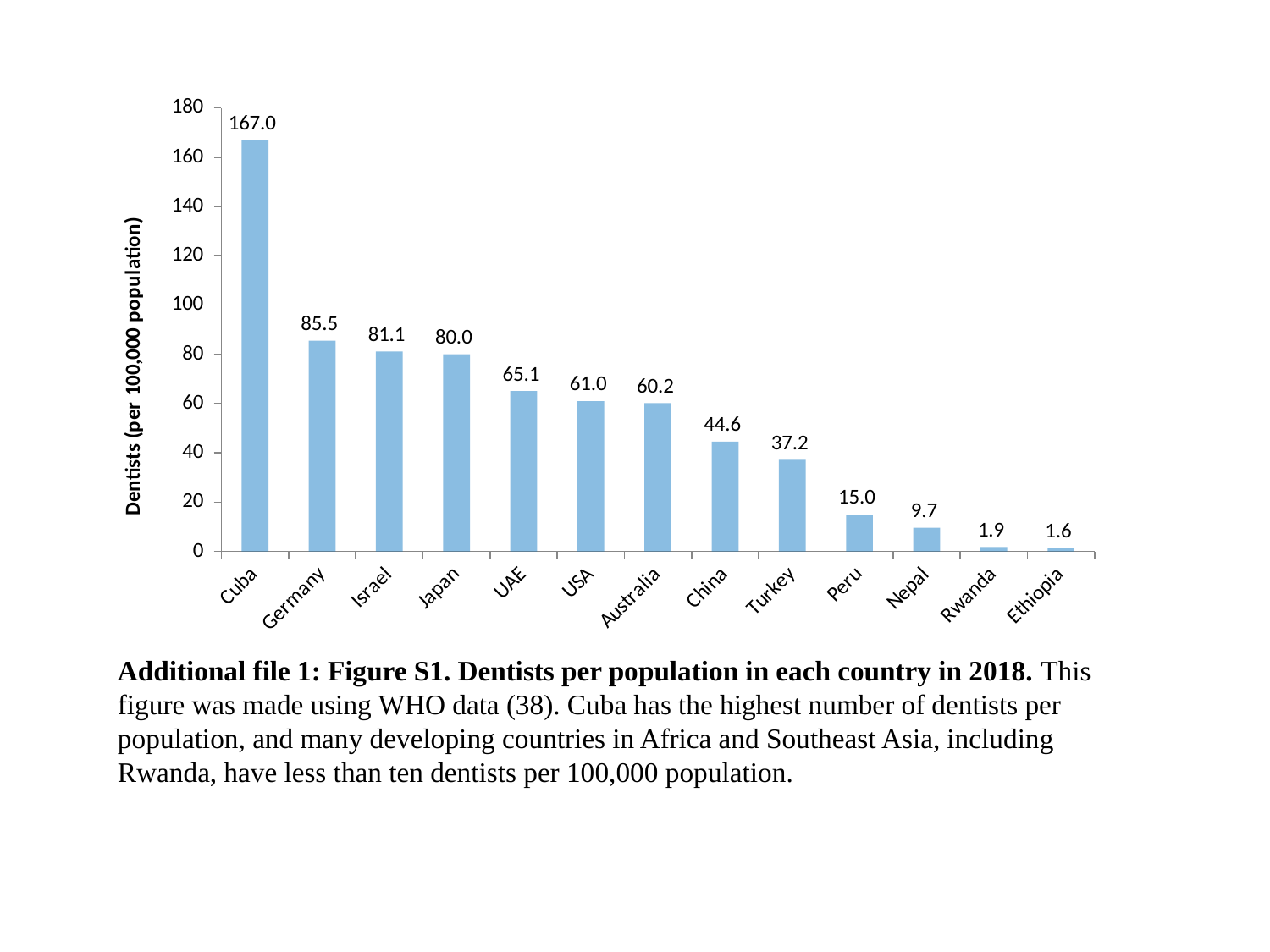

### Chart
| Category | Dentists (per 10 000 population) |
|---|---|
| Cuba | 166.99 |
| Germany | 85.53 |
| Israel | 81.13 |
| Japan | 80.03999999999999 |
| UAE | 65.13 |
| USA | 61.0 |
| Australia | 60.17 |
| China | 44.58 |
| Turkey | 37.18 |
| Peru | 15.0 |
| Nepal | 9.67 |
| Rwanda | 1.85 |
| Ethiopia | 1.6 |Additional file 1: Figure S1. Dentists per population in each country in 2018. This figure was made using WHO data (38). Cuba has the highest number of dentists per population, and many developing countries in Africa and Southeast Asia, including Rwanda, have less than ten dentists per 100,000 population.
